# Cost-effectiveness analysis of zolbetuximab plus mFOLFOX6 as the first-line treatment for CLDN18.2-positive, HER2-negative advanced gastric or Gastroesophageal Adenocarcinoma

**DOI:** 10.3389/fphar.2023.1238009

**Published:** 2023-08-31

**Authors:** Yufan Huang, Maojin You, Qundan Wu, Ruijia Chen

**Affiliations:** ^1^ Department of Pharmacy, Mindong Hospital Affiliated to Fujian Medical University, Ningde, Fujian, China; ^2^ Department of Pharmacy, Quanzhou Skin Disease Prevention and Treatment Hospital, Quanzhou, Fujian, China; ^3^ Department of Pharmacy, Mengchao Hepatobiliary Hospital of Fujian Medical University, Fuzhou, Fujian, China

**Keywords:** cost-effectiveness, zolbetuximab, CLDN18.2-positive, HER2-negative, gastric or gastroesophageal adenocarcinoma, first-line treatment

## Abstract

**Background:** The SPOTLIGHT trial demonstrated that zolbetuximab plus mFOLFOX6 (ZOL-FO) as a first-line regimen compared with placebo plus mFOLFOX6 (PLB-FO) conferred clinical benefits to patients with CLDN18.2-positive, HER2-negative advanced gastric or gastroesophageal junction (G/GEJ) adenocarcinoma. However, due to the high cost of zolbetuximab, whether ZOL-FO is cost-effective compared with PLB-FO is unclear. This study aimed to evaluate the cost-effectiveness of ZOL-FO as a first-line treatment option for CLDN18.2-positive, HER2-negative advanced G/GEJ adenocarcinoma from the perspective of the Chinese healthcare system.

**Methods:** Markov models with three different health states were developed to assess the cost-effectiveness of ZOL-FO as a first-line treatment option for CLDN18.2-positive, HER2-negative advanced G/GEJ adenocarcinoma. Clinical efficacy data were obtained from the SPOTLIGHT trial; the drug’s cost was calculated at national bid prices, and other costs and utility values were obtained from the published literature. Outcomes included total costs, quality-adjusted life years (QALYs), and incremental cost-effectiveness ratios (ICERs). The model’s robustness was verified using one-way sensitivity and probabilistic sensitivity analyses.

**Results:** The ZOL-FO group gained 1.64 QALYs at $87,746.35, while the PLB-FO group gained 1.23 QALYs at $11,947.81. The ICER for ZOL-FO *versus* PLB-FO was $185,353.28 per QALY gained. The parameters exerting an important impact on the model results were the price of zolbetuximab, body surface area, and progression-free survival utility. At a willingness-to-pay threshold of $38,201/QALY, ZOL-FO had a 0% probability of cost-effectiveness compared with PLB-FO.

**Conclusion:** From the perspective of the Chinese healthcare system, ZOL-FO is unlikely to be cost-effective as the first-line treatment option for CLDN18.2-positive, HER2-negative advanced G/GEJ adenocarcinoma.

## 1 Introduction

Gastric cancer (GC), with the fourth highest mortality rate and the fifth highest incidence among all malignant diseases, is a common cancer that threatens human health (Sung et al., 2021). China is at a high risk of GC, with more than 6.7 million newly diagnosed cases and approximately 5 million new deaths each year, accounting for 42% and 45% of the global cases, respectively (Chen et al., 2016). Nearly 90% of GC patients already develop metastases by the time they are first diagnosed (Zeng et al., 2018), and their prognoses are poor, with a 5-year survival rate of only 5% (Shu et al., 2022). The cancer of the gastroesophageal junction can also be classified as GC (Smyth et al., 2020). In gastric or gastroesophageal junction (G/GEJ) cancers, more than 90% of the histological types are adenocarcinomas (Ajani et al., 2017). The standard first-line treatment regimen for advanced G/GEJ adenocarcinoma is platinum combined with fluorouracil therapy (Wang et al., 2021); however, this chemotherapy has unsatisfactory efficacy, with a median survival of less than 1 year (Shitara et al., 2023). In recent years, although chemotherapy plus trastuzumab or nivolumab has been used as first-line treatment for advanced G/GEJ adenocarcinoma with HER-2-positive or high programmed death-ligand 1 (PD-L1) co-positive score, respectively (Bang et al., 2010; Jiang et al., 2022), the survival benefit remains low and the disease may rapidly recur or progress (Nakamura et al., 2021; Myer et al., 2022), necessitating the need to explore new molecular targets (Salati et al., 2023).

Claudin 18.2 (CLDN18.2), the tight junction protein, is a promising target for the treatment of G/GEJ adenocarcinoma (Sahin et al., 2008). Zolbetuximab, a chimeric IgG1 monoclonal antibody, targets and binds to CLDN18.2, thus inducing cell death in CLDN18.2-positive G/GEJ adenocarcinoma (Sahin et al., 2018). A recent phase III clinical trial (SPOTLIGHT) evaluated the efficacy and safety of zolbetuximab plus mFOLFOX6 (modified folinic acid, fluorouracil, and oxaliplatin regimen, ZOL-FO) as the first-line treatment of CLDN18.2-positive, HER2-negative locally advanced unresectable or metastatic G/GEJ adenocarcinoma (Shitara et al., 2023). The results showed that ZOL-FO significantly improved the overall survival (OS) and progression-free survival (PFS) of patients with CLDN18.2-positive, HER2-negative advanced G/GEJ adenocarcinoma compared with placebo plus mFOLFOX6 (PLB-FO), giving new hope for patients with advanced G/GEJ adenocarcinoma.

Although ZOL-FO provides clinical benefits for patients with CLDN18.2-positive, HER2-negative advanced G/GEJ adenocarcinoma, its high cost limits its widespread use. Therefore, the cost-effectiveness of ZOL-FO must be evaluated by pharmacoeconomic methods to assess the clinical benefits and potential financial consequences of ZOL-FO for patients with advanced G/GEJ adenocarcinoma and determine the rationale for its widespread use in the future. To the best of our knowledge, the economics of ZOL-FO has not been evaluated. This study estimates the cost-effectiveness of ZOL-FO as a first-line regimen for the treatment ofCLDN18.2-positive, HER2-negative advanced G/GEJ adenocarcinoma compared with PLB-FO from the perspective of the Chinese healthcare system based on the results obtained from the SPOTLIGHT trial (Shitara et al., 2023). This study was designed according to the Comprehensive Health Economic Assessment Reporting Standards 2022 (CHEERS 2022) (Husereau et al., 2022) (Supplementary Table SA).

## 2 Methods

### 2.1 Model construction

**FIGURE 1:**
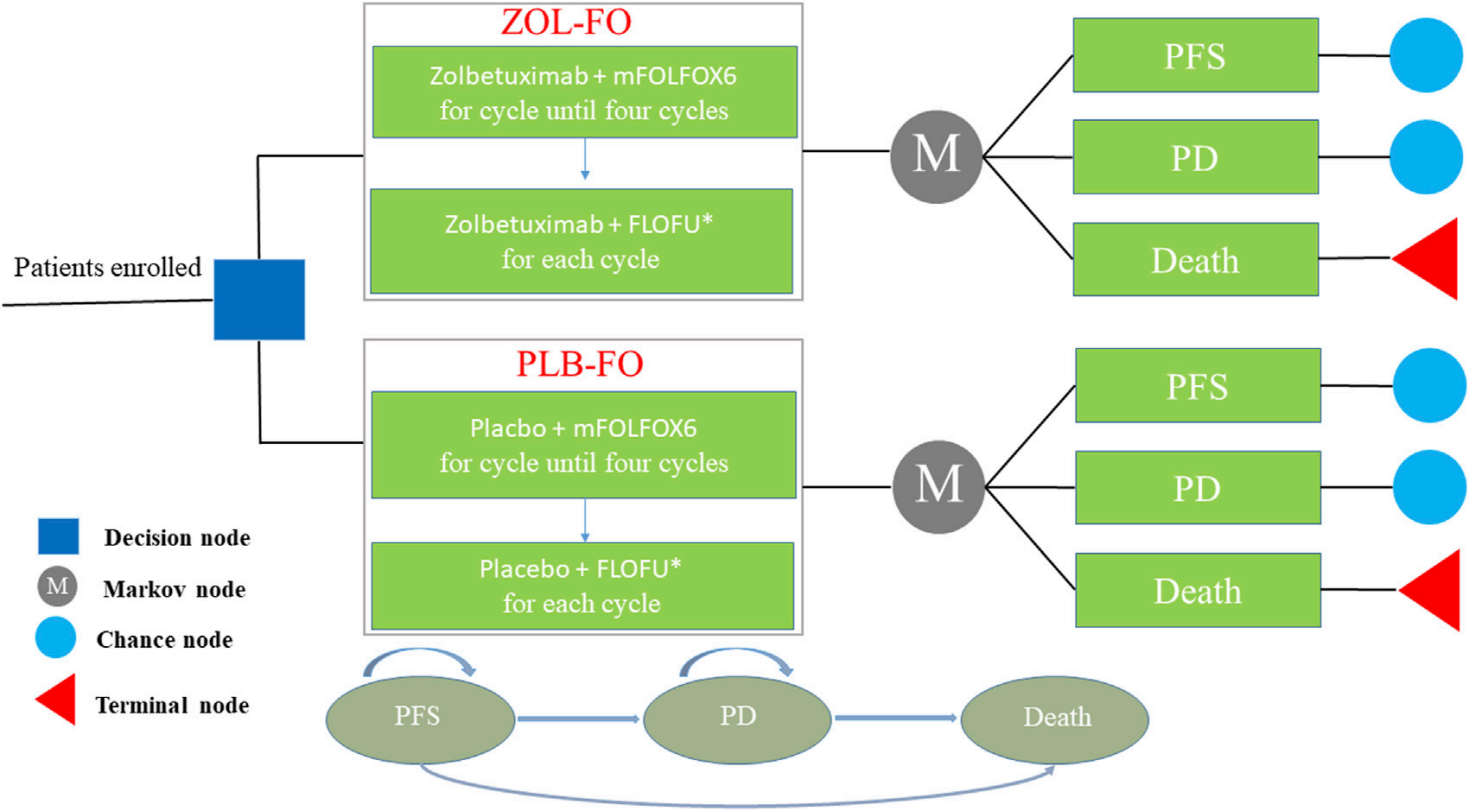
The Markov model simulating outcomes for the CAPSTONE-1 trial. All patients started with PFS state and received treatment with ZOL-FO or PLB-FO. FLOFU*, folinic acid and fluorouracil at the discretion of the investigator; PD, progressive disease; PFS, progression-free survival; PLB-FO, placebo plus mFOLFOX6; ZOL-FO, zolbetuximab plus mFOLFOX6.

Markov models were developed using TreeAge Pro 2022 (TreeAge Software, Williams-town, MA, United States) to estimate the cost and effectiveness of ZOL-FO compared with PLB-FO for patients withCLDN18.2-positive, HER2-negative advanced G/GEJ adenocarcinoma. The model contains three different health states, that is, PFS, progressive disease (PD), and death, which are mutually exclusive (Figure 1). We assumed that all patients entered the model with PFS and then as the Markov model was run, patients either remained in their current health state or progressed to a new health state but were not allowed to return to their previous health state. The length of each cycle in the model was 42 days. The model duration was 110 cycles (approximately 12.7 years), which was determined by the expected time to death kept at 99% of the hypothetical patients. The background mortality rate of China in 2022 was considered in the model (National Bureau of Statistics of China, 2023). The output of the model included total costs, quality-adjusted life years (QALYs), and incremental cost-effectiveness ratios (ICERs). According to the China Guidelines for Pharmacoeconomic Evaluations, we used three times China’s GDP *per capita* in 2022 ($38,201/QALY) as the willingness-to-pay (WTP) threshold, and if the ICER was below our predefined WTP threshold, the treatment option was considered cost-effective. Economic analyses were based on published randomized clinical trials and mathematical models. As a result, institutional review board or ethics committee approval was not necessary for this study.

### 2.2 Clinical data and transition probability

The survival benefit and safety data of our study were based on the results of the SPOTLIGHT trial (Shitara et al., 2023). Patients in this trial were distributed across 215 centers in 20 countries worldwide and had to meet the following criteria: 1) ≥18 years of age; 2) CLDN18.2 positive and HER2 negative; 3) previously untreated locally advanced unresectable or metastatic G/GEJ adenocarcinoma; 4) Eastern Cooperative Oncology Group performance status score of 0 or 1, and 5) adequate organ function.

These patients were randomly assigned to either the ZOL-FO or PLB-FO group; those in the ZOL-FO group received zolbetuximab 800 mg/m^2^ (cycle 1, day 1), followed by 600 mg/m^2^ (cycle 1, day 22, and days 1 and 22 of subsequent cycles), plus mFOLFOX6 (folinic acid 400 mg/m^2^; fluorouracil 2,800 mg/m^2^; oxaliplatin 85 mg/m^2^; days 1, 15, and 29 of each cycle). Patients in the PLB-FO group received a placebo plus mFOLFOX6. All patients receiving four cycles of treatment without disease progression continued zolbetuximab or placebo plus folinic acid and fluorouracil at the discretion of the investigator until disease progression or onset of toxic effects. Based on the SPOTLIGHT trial (Shitara et al., 2023), we assumed that when patients showed disease progression, a subset received chemotherapy, immunotherapy, or targeted therapy, and others received the best supportive care. All patients received the best supportive care after the failure of second-line therapy.

The transition probabilities between different health states were estimated based on the Kaplan-Meier survival curves from the SPOTLIGHT trial (Shitara et al., 2023). First, OS and PFS data points from Kaplan-Meier survival curves for both treatment groups were extracted using GetData Graph Digitizer (version 1.2), a software that digitizes images. Then, according to the method described by Guyot et al. (Guyot et al., 2012), the R software (version 4.2.0) was used to reconstruct Kaplan-Meier survival curves and extrapolate long-term clinical outcomes beyond the follow-up time, using the extracted data points. Various distribution functions, including exponential, Weibull, log-normal, and log-logistic, were assessed to identify the most suitable survival function based on the Akaike information criterion (AIC) and Bayesian information criterion (BIC). Lower AIC and BIC values indicated a better fit (Ishak et al., 2013; Williams et al., 2017). The AIC and BIC values for these distribution functions are presented in Supplementary Figure SB. Ultimately, the log-logistic distribution function was determined to best fit the PFS and OS data for both treatment groups (Table 1, Supplementary Figure SA). Accordingly, the time-dependent jump probability for each cycle in the model was calculated using the following equation: 1−{[1+λtγ]/[1+λ(t+1)γ]} (t, Current model cycle; λ, scale parameter; γ, shape parameter) (Diaby et al., 2014).

**TABLE 1:** Relevant parameters of the survival distribution.

Parameters	Value	Source
Loglogistic survival model of PFS		
** **PLB-FO	Scale = 0.1091424, Shape = 1.892484	Shitara et al. (2023)
** **ZOL-FO	Scale = 0.08578011, Shape = 1.596389	Shitara et al. (2023)
Log-logistic survival model of OS		
** **PLB-FO	Scale = 0.06858134, Shape = 1.943703	Shitara et al. (2023)
** **ZOL-FO	Scale = 0.05460772, Shape = 1.620319	Shitara et al. (2023)

OS, overall survival; PFS, progression-free survival; PLB-FO, placebo plus mFOLFOX6; ZOL-FO, zolbetuximab plus mFOLFOX6.

### 2.3 Costs and utilities

Only direct medical costs were considered, including costs of drugs, routine follow-up, best supportive care, tests, terminal care in end-of-life, and management of grade 3 or higher adverse reactions with an incidence greater than 5% considered (Table 2). The costs of these drugs were obtained from the national tender price. However, zolbetuximab is not yet available in the market, so we used the price of nivolumab in China, an immune checkpoint inhibitor recommended for first-line treatment of GC, as the reference price for zolbetuximab (converted to the cost needed for a single treatment), according to the method of Weng et al. (Weng et al., 2020). Other costs were obtained from published literature and were adjusted to costs in 2022 based on the China Medical Price Index (National Bureau of Statistics of China, 2023). All costs were converted to US dollars at the average US-China exchange rate in 2022 (1$ = 6.73 RMB). To calculate the dose administered to patients, we assumed that the patients had a body weight of 65 kg and a body surface area of 1.72 m^2^(Liu et al., 2022; Shu et al., 2022). The PFS and PD in this study were obtained from published Chinese literature because relevant quality-of-life data for patients were not available from the SPOTLIGHT trial (Table 2). To reduce the impact of using the same utility in the ZOL-FO and PLB-FO groups, we also considered the disutility of adverse reactions of grade 3 and above with an incidence of >5% in our model. We discounted the costs and health utilities at 5% per year according to the China Guidelines for Pharmacoeconomic Evaluations (Liu, 2020).

**TABLE 2:** Basic parameters of the input model and the range of sensitivity analyses.

Variable	Base value	Range		Distribution	Source
		Min	Max		
ZOL-FO group: Incidence of AEs (%)					
** **Nausea/Vomiting	32.26	25.81	38.71	Beta	Shitara et al. (2023)
** **Neutropenia	28.32	22.65	33.98	Beta	Shitara et al. (2023)
** **Anemia	8.60	6.88	10.32	Beta	Shitara et al. (2023)
** **Neutrophil count decrease	24.73	19.78	29.68	Beta	Shitara et al. (2023)
** **Fatigue	6.09	4.87	7.31	Beta	Shitara et al. (2023)
PLB-FO group: Incidence of AEs (%)					
** **Nausea/Vomiting	12.23	9.78	14.68	Beta	Shitara et al. (2023)
** **Neutropenia	23.38	18.71	28.06	Beta	Shitara et al. (2023)
** **Anemia	9.35	7.48	11.22	Beta	Shitara et al. (2023)
** **Neutrophil count decrease	24.82	19.86	29.78	Beta	Shitara et al. (2023)
** **Fatigue	5.04	4.03	6.04	Beta	Shitara et al. (2023)
Costs ($)					
** **Folinic acid (100 mg)	17.54	14.03	21.05	Gamma	Yao (2023)
** **Fluorouracil (100 mg)	1.78	1.42	2.14	Gamma	Yao (2023)
** **Oxaliplatin (100 mg)	59.82	47.86	71.78	Gamma	Yao (2023)
** **Zolbetuximab (100 mg)	258.20	206.56	309.84	Gamma	Yao (2023)
** **Paclitaxel (30 mg)	10.10	8.08	12.12	Gamma	Yao (2023)
** **Nivolumab (100 mg)	1374.44	1099.55	1649.33	Gamma	Yao (2023)
** **Best supportive care per cycle	182.23	145.78	218.68	Gamma	Zhang et al. (2021a)
** **Routine follow-up per cycle	73.72	58.98	88.46	Gamma	Zhang et al. (2021b)
** **Tests per cycle	357.34	285.87	428.81	Gamma	Liu et al. (2022)
** **Terminal care in end-of-life	1489.51	1191.60	1787.41	Gamma	Liu et al. (2023)
** **Nausea/Vomiting	101.15	80.92	121.38	Gamma	Zhan et al. (2022)
** **Neutropenia	454.26	363.41	545.11	Gamma	Liu et al. (2022)
** **Anemia	336.63	269.30	403.96	Gamma	Zhan et al. (2022)
** **Neutrophil count decrease	454.26	363.41	545.11	Gamma	Liu et al. (2022)
** **Fatigue	115.40	92.32	138.48	Gamma	Wu et al. (2012)
Utility value					
** **PFS	0.797	0.638	0.956	Beta	Shu et al. (2022)
** **PD	0.577	0.462	0.692	Beta	Shu et al. (2022)
Disutility due to AEs					
** **Nausea/Vomiting	−0.12	−0.10	−0.14	Beta	Nafees et al. (2017)
** **Neutropenia	−0.20	−0.16	−0.24	Beta	Nafees et al. (2017)
** **Anemia	−0.07	−0.06	−0.08	Beta	Cai et al. (2021)
** **Neutrophil count decrease	−0.20	−0.16	−0.24	Beta	Nafees et al. (2017)
** **Fatigue	−0.07	−0.06	−0.08	Beta	Nafees et al. (2017)
** **Discount rate	0.05	0.00	0.08	Fixed	Liu (2020)
** **Weight (kg)	65	52.00	78.00	Normal	Liu et al. (2022)
** **Body surface area (m^2^)	1.72	1.38	2.06	Normal	Liu et al. (2022)
The proportion of subsequent anticancer therapies (%)					
** **ZOL-FO group					
** **Chemotherapy	22.97	18.38	27.56	Beta	Shitara et al. (2023)
** **Targeted therapies	12.36	9.89	14.83	Beta	Shitara et al. (2023)
** **Immunotherapies	9.19	7.35	11.03	Beta	Shitara et al. (2023)
PLB-FO group					
** **Chemotherapy	24.11	19.29	28.93	Beta	Shitara et al. (2023)
** **Targeted therapies	12.06	9.65	14.47	Beta	Shitara et al. (2023)
** **Immunotherapies	9.93	7.94	11.92	Beta	Shitara et al. (2023)

AE, adverse event; PD, progressive disease; PFS, progression-free survival; PLB-FO, placebo plus mFOLFOX6; OS, overall survival; ZOL-FO, zolbetuximab plus mFOLFOX6.

### 2.4 Sensitivity analysis

To examine the model’s robustness and the uncertainty in the parameter estimates, we performed one-way and probabilistic sensitivity analyses. To perform a one-way sensitivity analysis, we adjusted each parameter within a given range (Table 2) to determine the effect of these changes on the ICER. The ranges of variation for all parameters were 95% confidence intervals from the literature and were assumed at ±20% of the baseline values in the absence of data. The lower and upper bounds of the discount rate were set at 0% and 8%, respectively. The results of the one-way sensitivity analysis are presented as tornado plots. We assigned all parameters to the appropriate distributions (Table 2) in the model and performed a probabilistic sensitivity analysis (PSA) with 1,000 Monte Carlo simulations to determine the effect of simultaneous changes in multiple parameters on the model results. The results of PSA are represented as scatter plots. We explored the effect of different prices on varying cost-effective results of ZOL-FO by continuously changing the price of zolbetuximab.

### 2.5 Subgroup analysis

To assess the impact of subgroups with different baseline characteristics on the model results, we performed exploratory subgroup analysis. Due to the lack of sufficient data for each subgroup that could be used for survival analysis, according to the method described by Hoyle et al. (Hoyle et al., 2010), to facilitate subgroup survival extrapolation, we let all subgroups in the PLB-FO group use the same PFS and OS survival functions (log-logistic survival model) and used the subgroup-specific hazard ratio provided by the SPOTLIGHT trial (Table 3) to calculate ICERs and cost-effectiveness acceptability probabilities for each subgroup.

**TABLE 3:** Results of subgroup analyses.

Subgroup	PFS HR (95% CI)	OS HR (95% CI)	ICER ($/QALY)	Cost-effectiveness probability
Age(years)				
** **≤65	0.77 (0.58–1.02)	0.74 (0.56–0.98)	207832.26	0
** **>65	0.71 (0.49–1.04)	0.76 (0.53–1.09)	186178.39	0
** **≤75	0.74 (0.59–0.93)	0.71 (0.57–0.90)	171802.56	0
** **>75	0.96 (0.39–2.34)	1.32 (0.58–3.00)	—	—
Sex				
** **Male	0.78 (0.59–1.02	0.76 (0.58–1.00)	225438.13	0
** **Female	0.71 (0.49–1.03)	0.73 (0.50–1.05)	199977.43	0
Region				
** **Asia	0.56 (0.37–0.85)	0.64 (0.44–0.95)	154637.71	0
** **Non-Asia	0.85 (0.65–1.11)	0.80 (0.61–1.04)	274414.41	0
Number of metastatic sites				
0–2	0.73 (0.56–0.94)	0.77 (0.59–0.99)	232964.07	0
≥3	0.84 (0.55–1.30)	0.67 (0.44–1.03)	214541.33	0
Previous gastrectomy				
** **No	0.81 (0.62–1.05)	0.84 (0.65–1.09)	566161.28	0
** **Yes	0.62 (0.41–0.94)	0.58 (0.38–0.87)	106015.93	0.20%
Primary site				
** **Stomach	0.69 (0.53–0.89)	0.67 (0.52–0.86)	163375.03	0
** **Gastro-oesophageal junction	1.02 (0.65–1.59)	1.07 (0.69–1.67)	—	—
Lauren classification				
** **Diffuse	0.76 (0.51–1.13)	0.77 (0.53–1.11)	234055.32	0
** **Intestinal	0.58 (0.38–0.89)	0.55 (0.36–0.85)	119418.99	0
** **Mixed or other	0.93 (0.60–1.43)	0.99 (0.64–1.54)	2792896.79	0
Country				
** **Japan	0.48 (0.23–1.01)	0.71 (0.41–1.25)	188331.92	0
** **Non-Japan	0.79 (0.63–1.00)	0.76 (0.60–0.96)	225670.02	0
** **China	0.50 (0.20–1.26)	0.91 (0.36–2.32)	198642.25	0
** **Non-China	0.75 (0.60–0.95)	0.74 (0.59–0.92)	207699.78	0
Race				
** **White	0.93 (0.68–1.27)	0.95 (0.70–1.29)	1051758.78	0
** **Asian	0.53 (0.35–0.79)	0.57 (0.39–0.83)	129440.25	0
Tobacco history				
** **Never	0.74 (0.54–1.01)	0.68 (0.49–0.93)	167176.02	0
** **Current	1.00 (0.48–2.09)	0.82 (0.40–1.69)	—	—
** **Former	0.71 (0.50–1.02)	0.81 (0.58–1.13)	273231.69	0

HR, hazard ratio; ICER, incremental cost-effectiveness ratio; OS, overall survival; PFS, progression-free survival.

## 3 Results

### 3.1 Base case analysis

Our findings are expressed in terms of the total costs, QALYs, and ICERs (Table 4); 1.64 QALYs were achieved in the ZOL-FO group for $87,746.35. In the PLB-FO group, the survival benefit was 1.23 QALYs with an investment of $11,947.81. Compared with the PLB-FO, the mean incremental effectiveness and cost in the ZOL-FO were 0.41 QALYs and $75,798.54, respectively. The ICER for ZOL-FO *versus* PLB-FO was $185,353.28 per QALY gained. Therefore, in China, ZOL-FO is unlikely to be a cost-effective first-line treatment strategy for CLDN18.2-positive, HER2-negative advanced G/GEJ adenocarcinoma compared with PLB-FO at a WTP threshold of $38,201/QALY.

**TABLE 4:** The cost and outcome results of the cost-effectiveness analysis.

Regimen	ZOL-FO	PLB-FO	Incremental
Total QALYs	1.64	1.23	0.41
Total costs, $	87746.35	11947.81	75798.54
ICER, $ Per QALY	—	—	185353.28

ICER, incremental cost-effectiveness ratio; PLB-FO, placebo plus mFOLFOX6; QALY, quality-adjusted life year; ZOL-FO, Zolbetuximab plus mFOLFOX6.

### 3.2 Sensitivity analysis

**FIGURE 2:**
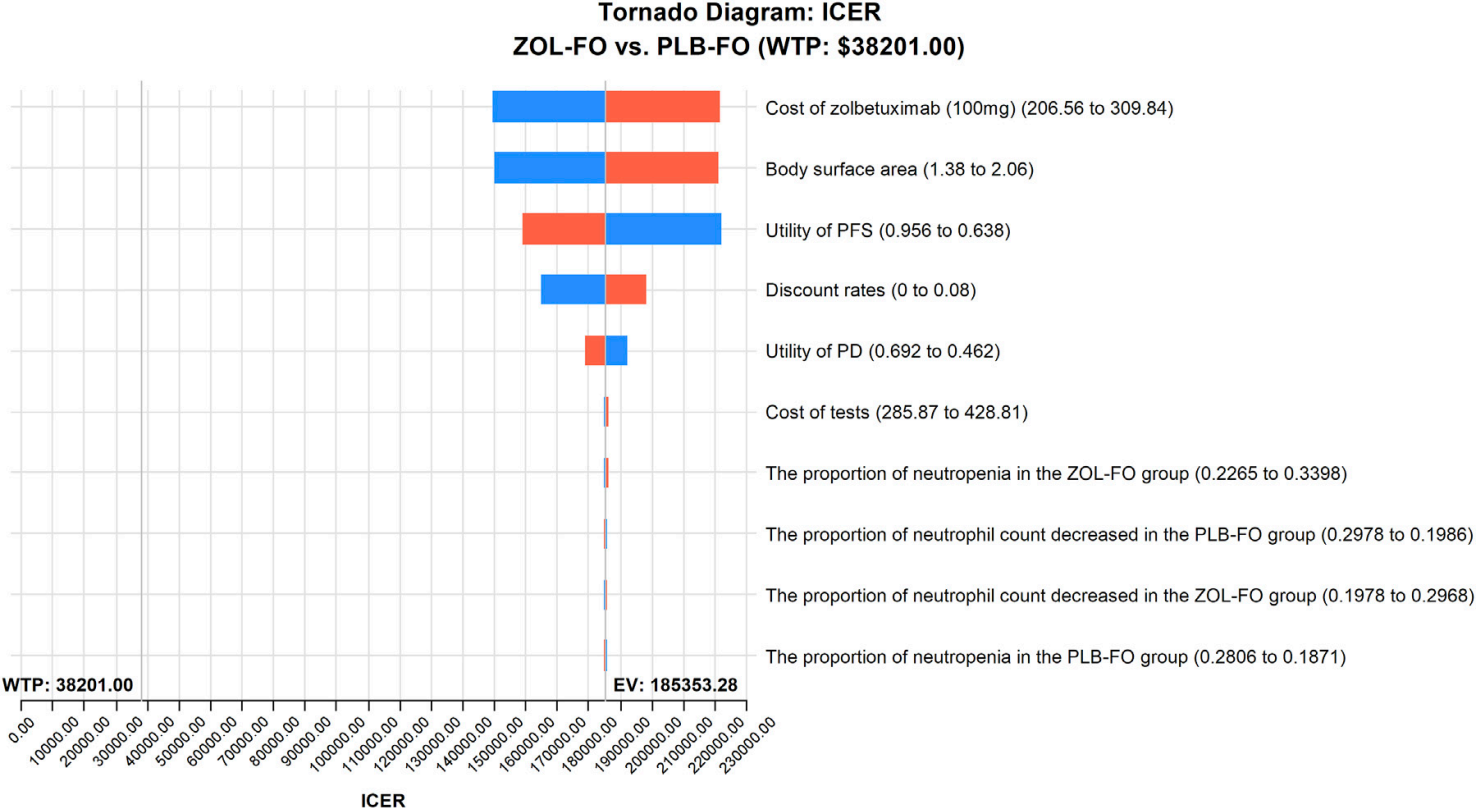
One-way sensitivity analyses of ZOL-FO in comparison with PLB-FO. ICER, incremental cost-effectiveness ratio; PD, progressive disease; PFS, progression-free survival; PLB-FO, placebo plus mFOLFOX6; ZOL-FO, zolbetuximab plus mFOLFOX6.

**FIGURE 3:**
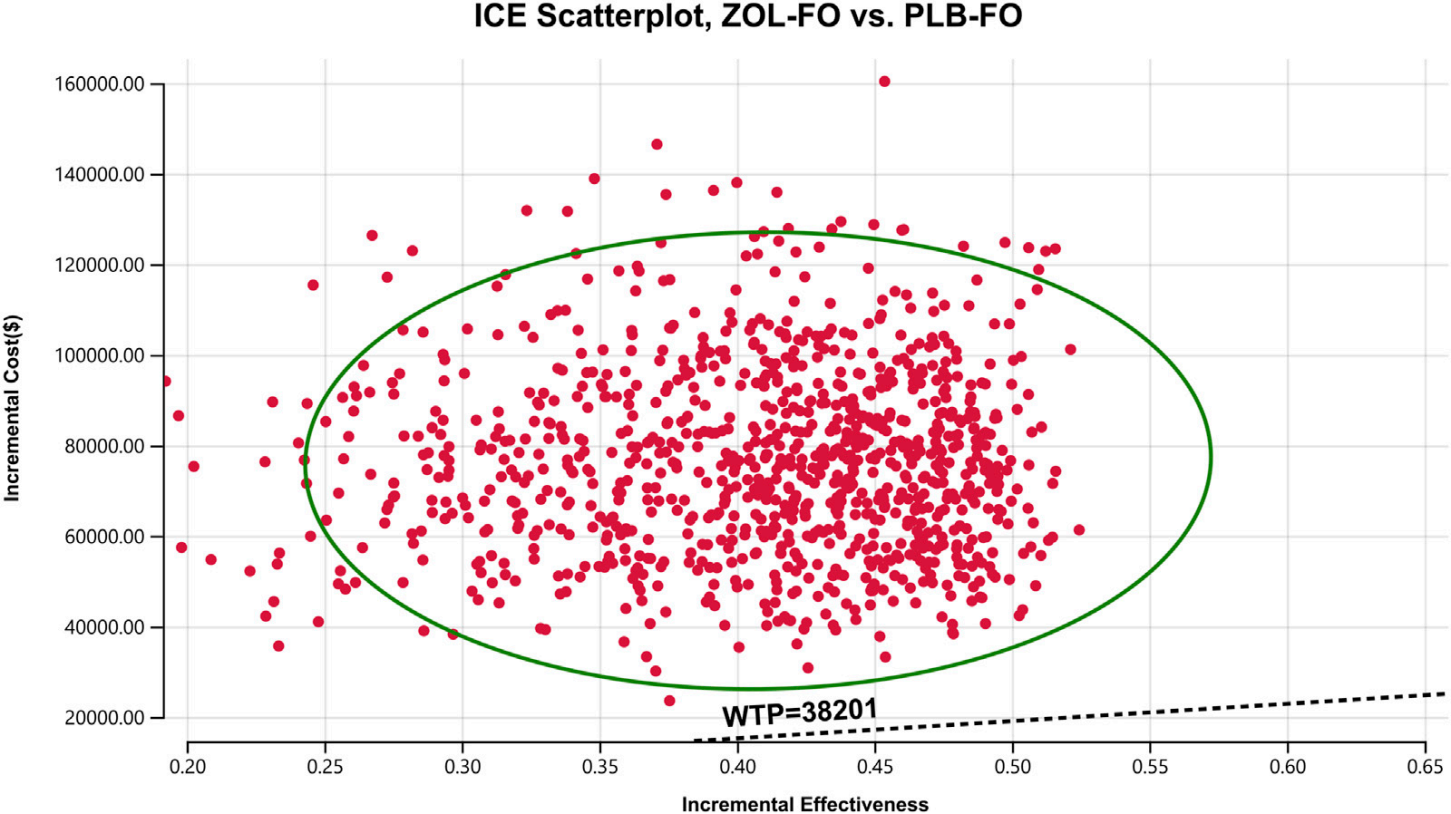
A probabilistic scatter plot of the ICER between the ZOL-FO group and the PLB-FO group. Each point means the ICER for 1 simulation. Ellipses are used to indicate 95% confidence intervals. Points that lie below the ICER threshold represent cost-effective simulations. ICER, incremental cost-effectiveness; PLB-FO, placebo plus mFOLFOX6; WTP, willingness-to-pay; ZOL-FO, zolbetuximab plus mFOLFOX6.

The results of the one-way sensitivity analysis showed that in the tornado plot (Figure 2), the most important parameters that affected the model results were zolbetuximab’s price, body surface area, and PFS utility. However, despite changing the values of these parameters, the ICER was always above our predetermined WTP threshold, implying that changes in parameter values could not change our model results. The variables having less impact on the results included the discount rate, PD utility, and the cost per cycle of tests. The PSA results are represented as scatter plots (Figure 3), and when the WTP threshold is $38,201/QALY, the probability that ZOL-FO is cost-effective compared to PLB-FO is 0%. When zolbetuximab’s price (100 mg) drops below 18.33% of the predetermined price, i.e., below $43.72, ZOL-FO will be a cost-effective first-line treatment option for CLDN18.2-positive, HER2-negative advanced G/GEJ adenocarcinoma.

### 3.3 Subgroup analysis

Compared with the PLB-FO group, all subgroups in the ZOL-FO group had ICERs above the WTP threshold of $38,201/QALY, with 0% probability of cost-effectiveness, except for the previous gastrectomy subgroup which had 0.2% (Table 3). Notably, in the PLB-FO group, more benefits and fewer costs were found for the subgroup with age >75 years, gastro-oesophageal junction cancer, and the current tobacco history, suggesting that these subgroups were not likely to be cost-effective with the ZOL-FO regimen; it is important to interpret these results cautiously due to the limited sample enrollment.

## 4 Discussion

In the first-line treatment of advanced HER2-negative GC, the American Society of Clinical Oncology recommends the use of nivolumab plus chemotherapy for patients with PD-L1 CPS (Combined Positive Score) ≥5 in G/GEJ adenocarcinoma, and pembrolizumab plus chemotherapy for patients with PD-L1 CPS ≥10 in GEJ adenocarcinoma(Press et al., 2017; Shah et al., 2023) CLDN18.2 is expressed in most G/GEJ adenocarcinoma cells (Shitara et al., 2023). The SPOTLIGHT trial evaluated the efficacy and safety of ZOL-FO as a first-line regimen for the treatment of CLDN18.2-positive, HER2-negative advanced G/GEJ adenocarcinoma (Shitara et al., 2023). The trial found that ZOL-FO significantly prolonged OS [median OS, 18.23 vs 15.54 months, HR0.75(95%CI 0.60–0.94)] and PFS [median PFS, 10.61 vs 8.67 months, HR0.75(95%CI 0.60–0.94)] in CLDN18.2-positive, HER2-negative advanced G/GEJ adenocarcinoma compared with PLB-FO in safely and manageably, providing a new first-line treatment option for advanced G/GEJ adenocarcinoma. The results of the SPOTLIGHT trial are expected to drive the widespread use of zolbetuximab for treating CLDN18.2-positive, HER2-negative advanced G/GEJ adenocarcinoma patients, leading to a significant increase in economic burden that will certainly become an important issue for healthcare decision-makers. Therefore, an economic evaluation of zolbetuximab is imperative.

To our knowledge, this study is the first to evaluate the cost-effectiveness of ZOL-FO as the first-line treatment option for patients with CLDN18.2-positive, HER2-negative advanced G/GEJ adenocarcinoma, and its results will be instructive in China and other countries, which is the most important innovative point of this study. The results of this study show that ZOL-FO costs an additional $185,353.28 per additional QALY provided compared with PLB-FO, much higher than our predetermined WTP ($38,201/QALY). Thus, ZOL-FO for CLDN18.2-positive, HER2-negative advanced G/GEJ adenocarcinoma is not cost-effective in China. Zolbetuximab costs much more than a placebo but does not provide a sufficient incremental survival benefit, which is the main reason it is not cost-effective. The results of the subgroup analysis also support that ZOL-FO is not a cost-effective treatment option. However, the results of this study should not be a reason to restrict the use of zolbetuximab, as it may result in a missed opportunity for beneficial treatment but should be considered as an economic reference for the country when negotiating drug prices (Yue et al., 2021). One-way sensitivity analysis also showed that zolbetuximab’s cost was the most important factor affecting the model results. We, therefore, have made adjustments to the price of zolbetuximab to obtain different cost-effective results. ZOL-FO was cost-effective only when zolbetuximab (100 mg) was below $47.32.

Since 2018, the national health insurance administration has conducted several rounds of price negotiations with drug manufacturers for anti-cancer drugs, aiming to reduce the economic burden of cancer patients and society. The price of many anticancer drugs has been reduced by approximately 70% (Zhang Q. et al., 2021; Zhang et al., 2022). As of December 2022, China has approved the market launch of 16 immune checkpoint inhibitors (NMPA, 2023). In tertiary hospitals, the reimbursement rate for medical expenses of patients with medical insurance is approximately 70%, while primary healthcare institutions tend to offer an even higher reimbursement rate(Qin et al., 2023). These measures have significantly enhanced accessibility and affordability for patients. The results of this study are expected to provide the national health insurance administration with an economic reference for post-marketing price negotiations for zolbetuximab. We also recommend that manufacturers implement medication assistance programs after patients have completed a certain treatment cycle to enhance the accessibility of medications for patients.

Many antineoplastic drugs are considered uneconomical due to their small incremental survival benefit and high incremental cost for advanced GC (Shu et al., 2022). The results of Shu et al. (Shu et al., 2022) and Jiang et al. (Jiang et al., 2022) showed that nivolumab plus chemotherapy was not cost-effective as a first-line treatment for advanced gastric/gastroesophageal junction/esophageal adenocarcinoma compared with chemotherapy alone in China. The results of Li et al. (Li et al., 2020) suggest that for Chinese patients with advanced GC, second-line adjuvant therapy with ramucirumab combined with paclitaxel is unlikely to be cost-effective in a reasonable and expected range of drug costs. Chen et al. (Chen et al., 2017) suggest that apatinib is not cost-effective as third-line therapy for advanced GC in China. These are consistent with the results of our study.

Focusing solely on the cost-effectiveness evaluation of the treatment regimen from the perspective of China’s healthcare system may lead to an underestimation of ZOL-FO’s cost-effectiveness. As we know, China is classified as a developing country, and its *per capita* GDP is significantly lower compared to developed countries in Europe and America. In developed countries, the higher average income enables patients to more easily bear treatment costs, and medical insurance coverage is often more extensive. These factors may result in more widespread adoption of ZOL-FO in those countries, leading to a more positive impact on patients’ treatment outcomes. Therefore, when evaluating the cost-effectiveness of ZOL-FO, it is essential to consider the economic conditions and disparities in healthcare systems among different countries. Furthermore, it is important to recognize the ethical issues of recommending expensive drugs to patients in oncology that have little to no clinical benefit. The occurrence of such situations is indeed regrettable and calls for further ethical and societal discussions to address them.

Our findings have other important advantages. First, ZOL-FO and PLB-FO were directly compared in the SPOTLIGHT trial, and our study used 4-year survival data from the recently published SPOTLIGHT trial. Second, 31% of the patients enrolled in the SPOTLIGHT trial were from Asia, so the results of the SPOTLIGHT trial can be extrapolated to a large extent to the Chinese population. Third, the economic outcomes of the 26 subgroups defined in the SPOTLIGHT trial were examined in this study, and physicians, patients, and policymakers may benefit from economic information about these subgroups.

However, our study has some limitations. First, due to practical limitations, we were unable to obtain long-term survival data, and a log-logistic survival model was used in this study to simulate data beyond the follow-up time frame, thus likely deviating from the real data. Second, we assumed that patients received the best supportive care at the time of disease progression, except for a subset treated with chemotherapy, targeted therapy, and immunotherapy, which may not accurately reflect the actual clinical situation. Third, only adverse events of grade 3 or higher with an incidence of >5% were included in the model. However, the results of the sensitivity analysis showed that changes in the incidence of adverse events did not significantly affect our results. Fourth, although we performed subgroup analyses, we should interpret this result with caution due to the small number of patients in the subgroup. Finally, the SPOTLIGHT trial did not provide data on quality of life, and the survival utility values in this study were derived from published literature in China, which may have led to bias in the model results but sensitivity analysis showed that our model was robust.

## 5 Conclusion

This study is the first to evaluate the cost-effectiveness of ZOL-FO from the perspective of the Chinese healthcare system using the results of recent clinical trials. Our results suggest ZOL-FO is not cost-effective as the first-line treatment for CLDN18.2-positive, HER2-negative advanced G/GEJ adenocarcinoma compared with PLB-FO.

## Data Availability

The original contributions presented in the study are included in the article/Supplementary Material further inquiries can be directed to the corresponding author.
