# Supplementary material for: Cost-effectiveness analysis of zolbetuximab plus mFOLFOX6 as the first-line treatment for CLDN18.2-positive, HER2-negative advanced gastric or Gastroesophageal Adenocarcinoma
Source: Front Pharmacol. 2023 Aug 31;14:1238009. doi: 10.3389/fphar.2023.1238009 (PMC10500349; doi:10.3389/fphar.2023.1238009)
Supplement: Supplementary file 1 [file Table1.docx]

Supplementary Material

**Cost-Effectiveness Analysis of Zolbetuximab plus mFOLFOX6 as the First-Line Treatment for CLDN18.2-positive, HER2-negative Advanced** **Gastric or Gastroesophageal Adenocarcinoma**

**1. Supplementary Table A. CHEERS 2022 Checklist.**

**2. Supplementary Table B. Comparison of survival models.**

**3. Supplementary Figure A. Results of the survival curve fit the ZOL-FO group and PLB-FO group.**

**1.** **Supplementary Table A. CHEERS 2022 Checklist.**

| **Topic** | **No.** | **Item** | **Reported** |
| --- | --- | --- | --- |
| **Title** |  |  |  |
|  | 1 | Identify the study as an economic evaluation and specify the interventions being compared. | Yes |
| **Abstract** |  |  |  |
|  | 2 | Provide a structured summary that highlights context, key methods, results, and alternative analyses. | Yes |
| **Introduction** |  |  |  |
| **Background and objectives** | 3 | Give the context for the study, the study question, and its practical relevance for decision making in policy or practice. | Yes |
| **Methods** |  |  |  |
| **Health economic analysis plan** | 4 | Indicate whether a health economic analysis plan was developed and where available. | Yes |
| **Study population** | 5 | Describe characteristics of the study population (such as age range, demographics, socioeconomic, or clinical characteristics). | Yes |
| **Setting and location** | 6 | Provide relevant contextual information that may influence findings. | Yes |
| **Comparators** | 7 | Describe the interventions or strategies being compared and why chosen. | Yes |
| **Perspective** | 8 | State the perspective(s) adopted by the study and why chosen. | Yes |
| **Time horizon** | 9 | State the time horizon for the study and why appropriate. | Yes |
| **Discount rate** | 10 | Report the discount rate(s) and reason chosen. | Yes |
| **Selection of outcomes** | 11 | Describe what outcomes were used as the measure(s) of benefit(s) and harm(s). | Yes |
| **Measurement of outcomes** | 12 | Describe how outcomes used to capture benefit(s) and harm(s) were measured. | Yes |
| **Valuation of outcomes** | 13 | Describe the population and methods used to measure and value outcomes. | Yes |
| **Measurement and valuation of resources and costs** | 14 | Describe how costs were valued. | Yes |
| **Currency, price date, and conversion** | 15 | Report the dates of the estimated resource quantities and unit costs, plus the currency and year of conversion. | Yes |
| **Rationale and description of model** | 16 | If modelling is used, describe in detail and why used. Report if the model is publicly available and where it can be accessed. | Yes |
| **Analytics and assumptions** | 17 | Describe any methods for analysing or statistically transforming data, any extrapolation methods, and approaches for validating any model used. | Yes |
| **Characterising heterogeneity** | 18 | Describe any methods used for estimating how the results of the study vary for subgroups. | Yes |
| **Characterising distributional effects** | 19 | Describe how impacts are distributed across different individuals or adjustments made to reflect priority populations. | Yes |
| **Characterising uncertainty** | 20 | Describe methods to characterise any sources of uncertainty in the analysis. | Yes |
| **Approach to engagement with patients and others affected by the study** | 21 | Describe any approaches to engage patients or service recipients, the general public, communities, or stakeholders (such as clinicians or payers) in the design of the study. | Yes |
| **Results** |  |  |  |
| **Study parameters** | 22 | Report all analytic inputs (such as values, ranges, references) including uncertainty or distributional assumptions. | Yes |
| **Summary of main results** | 23 | Report the mean values for the main categories of costs and outcomes of interest and summarise them in the most appropriate overall measure. | Yes |
| **Effect of uncertainty** | 24 | Describe how uncertainty about analytic judgments, inputs, or projections affect findings. Report the effect of choice of discount rate and time horizon, if applicable. | Yes |
| **Effect of engagement with patients and others affected by the study** | 25 | Report on any difference patient/service recipient, general public, community, or stakeholder involvement made to the approach or findings of the study | Yes |
| **Discussion** |  |  |  |
| **Study findings, limitations, generalisability, and current knowledge** | 26 | Report key findings, limitations, ethical or equity considerations not captured, and how these could affect patients, policy, or practice. | Yes |
| **Other relevant information** |  |  |  |
| **Source of funding** | 27 | Describe how the study was funded and any role of the funder in the identification, design, conduct, and reporting of the analysis | Yes |
| **Conflicts of interest** | 28 | Report authors conflicts of interest according to journal or International Committee of Medical Journal Editors requirements. | Yes |

**2.** **Supplementary Table B. Comparison of survival models.**

|  | AIC | | BIC | |
| --- | --- | --- | --- | --- |
|  | ZOL-FO | PLB-FO | ZOL-FO | PLB-FO |
| OS |  |  |  |  |
| Exponential | 1386.734 | 1432.597 | 1386.731 | 1436.251 |
| Weibull | 1273.568 | 1398.969 | 1281.114 | 1406.425 |
| Log-normal | 1287.651 | 1419.023 | 1294.886 | 1426.267 |
| Log-logistic | 1276.629 | 1403.273 | 1283.567 | 1410.257 |
| PFS |  |  |  |  |
| Exponential | 1267.803 | 1219.495 | 1267.787 | 1223.158 |
| Weibull | 1141.857 | 1198.879 | 1149.253 | 1206.356 |
| Log-normal | 1146.052 | 1194.122 | 1153.288 | 1201.355 |
| Log-logistic | 1131.380 | 1186.274 | 1138.312 | 1193.212 |

AIC, Akaike information criterion; BIC, Bayesian Information Criterion; OS, overall survival; PFS, progression-free survival; PLB-FO, placebo plus mFOLFOX6; ZOL-FO, zolbetuximab plus mFOLFOX6.

**3.** **Supplementary Figure A. Results of the survival curve fit the ZOL-FO group and PLB-FO group.**


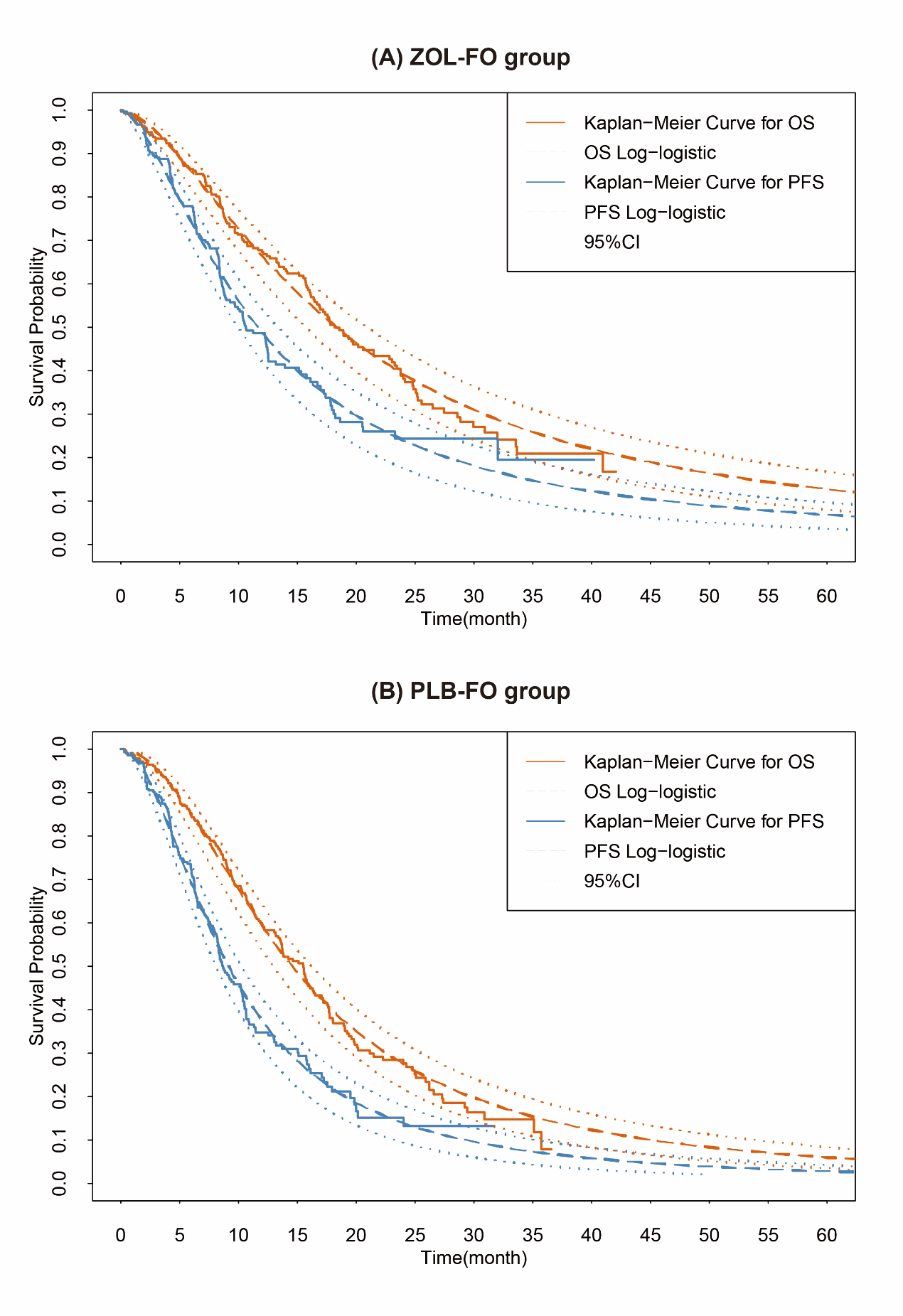


95%CI: 95% confidence interval; OS: Overall survival; PFS, progression-free survival; PLB-FO, placebo plus mFOLFOX6; ZOL-FO, zolbetuximab plus mFOLFOX6.
